# Executive Function, Language, and the Toddler’s Discovery of Representational Drawing

**DOI:** 10.3389/fpsyg.2021.659569

**Published:** 2021-06-03

**Authors:** Sabrina Panesi, Sergio Morra

**Affiliations:** ^1^DiSFor (Department of Education), Università di Genova, Genoa, Italy; ^2^National Research Council of Italy, Institute for Educational Technology, Genoa, Italy

**Keywords:** drawing, language, working memory, inhibition, shifting, executive function, toddlers, cognitive development

## Abstract

Working memory capacity and executive functions play important roles in the early development of drawing and language, but we lack models that specify the relationships among these representational systems and cognitive functions in toddlers. To respond to this need, the present study investigated the relations between drawing and language in very young children, and the role of working memory capacity, inhibition, and shifting in the association between these two representational systems. The participants were 80 children, 25–37 months old. The results revealed that in toddlers (a) all the measures of working memory, inhibition, and shifting loaded on a single factor of general executive functioning; (b) language and drawing are two distinct, but substantially correlated, representational systems; and (c) the development of executive function has a strong impact on language development, which in turn influences the development of drawing.

## Introduction

The 3rd year of life is a time of rapid development of symbolic representations including language, drawing, and symbolic play (Piaget, 1945/1962; Callaghan, 2000; DeLoache, 2004; Homer and Nelson, 2005; Callaghan and Corbit, 2014). Recent research indicates that working memory and executive functioning have an important role in the development of language (e.g., Ibbotson and Kearvell-White, 2015; Woodward et al., 2016; Gandolfi and Viterbori, 2020) and drawing (e.g., Morra, 2008a; Riggs et al., 2013; Morra and Panesi, 2017). However, much work is still needed to investigate the developmental relations among different aspects of cognition, such as the possible association between drawing and language and the possible influence of domain-general aspects of cognition (e.g., working memory capacity and executive functions) on the relation between language and drawing in very young children.^1^ Investigating these relations is theoretically very important because, after the crisis of Piaget’s theory as the dominant paradigm in cognitive development, research in the field has become quite fragmented. For instance, Siegler notes that, having dismissed a unified, encompassing theory such as Piaget’s and moving on to newer theories, “we have traded a rough, sometimes inaccurate depiction of the forest for innumerable, more accurate depictions of specific trees (and often their branches, twigs, leaves, and chloroplasts).” Siegler (2016, p.5) also suggests that the integration of well-grounded domain-specific theories should provide a basis for new, unified, general theories of development. As another example, Pascual-Leone and Johnson (2021) present an encompassing theory of cognitive development, essentially focused on the development of representations endowed with meaning and on the role of attentional systems. Indeed, the importance of representational systems in cognitive development has long been recognized (e.g., Case, 1985; Pratt and Garton, 1993); however, perhaps because of fragmentation of the field, the connections between different representational systems are still under-researched, and the role of working memory and executive function has only been studied separately in each representational domain.

Only few studies focused on the relationship between the early development of drawing and language as representational systems (Callaghan and Rankin, 2002; Toomela, 2002). Comparing the emergence of graphic symbolism and language, we can consider that the first words appear around the age of 1 year, whereas graphic symbol production emerges later, around 3 years of age (Golomb, 1981). Language production requires a well-developed vocal apparatus; infants are capable of making a range of language-like sounds early in their 1st year. In contrast, production in the graphic domain requires eye-hand coordination and the fine-tuned pincer grasp necessary to manipulate the tools of drawing, both of which are not refined enough until the 2nd year of life (Callaghan and Rankin, 2002). This could be one reason for the later emergence of drawing with respect to language. However, Callaghan (1999) also noted that 2-year olds failed to understand the symbolic nature of pictures. Thus, although children comprehend pictures before they can produce them, also picture comprehension occurs later than language comprehension. For instance, Adamson (1995) points out that language comprehension starts around 9 or 10 months of age, and language production starts around the first half of the 2nd year.

Panesi and Morra (2018) reviewed the literature on the relation between drawing and language development and considered a potential controversy among four theoretical accounts. First, Piaget (1945/1962) and Piaget and Inhelder (1969) regarded language and drawing as two expressions of the same, domain-general symbolic function that manifests itself in several signifying systems. Second, an opposite position argued for domain specificity; for instance, Chomsky (1964) claimed that language is based on specific, innate mechanisms, different from those involved in motor skill development. Third, drawing can be seen as a form of language. This view was expressed by Willats (1985), who theorized on “denotation systems”; the child uses picture primitives, such as dots, lines, and areas, to construct meaningful drawing schemes, which in turn can be combined according to syntactic rules of adjacency, occlusion, and projection. Similarly, Cohn (2012) suggested that drawing involves a lexicon of visual items, and outlines a parallel between language (where the lexical items are phonemes, morphemes, words, idioms, and perhaps whole sentences) and drawing (where the vocabulary includes simple graphemes and parts of images); these visual lexical items are combined in larger pictures, like words are combined in sentences. A fourth view suggests that drawing is influenced by language. For instance, the use of words as verbal labels could facilitate the transition from scribbling to the attribution of a meaning to more or less recognizable shapes (Callaghan, 2000; Toomela, 2002). Furthermore, verbal planning can be instrumental to the deployment of drawing activity (Freeman, 1972; Van Sommers, 1984; Golomb, 1992). Given the existence of different and sometimes contrasting views, more research is needed to clarify the relation between drawing and language. In particular, it seems necessary to test whether language development has a causal effect on drawing development and to what extent the development of working memory and executive control contributes to the development of both representational systems.

The question on the role of working memory and executive functions seems very important, because it concerns the role of domain-general cognitive functions in the development of specific cognitive domains, which has been a major issue in the cognitive developmental debates during the last 50 years. In particular, it is conceivable that these domain-general functions partly account for the developmental relation between drawing and language, because they seem to underpin development in both representational domains. In Piaget’s theory, the coordination of several sensorimotor schemes is essential to the emergence of intelligent problem-solving and symbolic representation and, according to neo-Piagetians (e.g., Case, 1985; Pascual-Leone and Johnson, 2005), the development of working memory capacity is essential to the coordination of schemes that enables the child’s acquisition of symbolization. However, research on the relations between language, drawing, and domain-general cognitive functions is still rather fragmentary.

Extensive research demonstrated the relevance of working memory capacity and executive functions to preschoolers’ and schoolchildren’s drawing (see Morra, 2008a,b; Panesi and Morra, 2016), but only few studies examined their role in the transition from scribbling to drawing. Morra and Panesi (2017) suggested that limited working memory or attentional capacity constrains the development of graphical abilities (and, more generally, cognitive abilities). During infancy, the coordination of increasing numbers of sensorimotor schemes places increasing loads on working memory. In particular, uncontrolled scribbling, typical at 18 months, requires coordinating five sensorimotor schemes; the subsequent transition to controlled scribbling, and then the production of forms, “diagrams” and “diagram aggregates” (as defined by Kellogg, 1969) would require additional schemes to be coordinated. In particular, a combination or aggregate of forms or diagrams seems to require that each single shape comprised in the combination be planned before drawing it. Furthermore, at some point in development, when scribbling is (at least) visually controlled, children start to attribute meaning to their scribbling activity (action representation; see Matthews, 1984) or to the form of scribbles (e.g., Adi-Japha et al., 1998). To do so, children need an additional scheme, i.e., the meaning attached to the current action or scribble, in addition to those required for controlled scribbling. However, toddlers most often attribute meaning to a scribble after having completed it, based on the current visual input and the memory of their own actions (Adi-Japha et al., 1998; see also Allen et al., 2016). If meaning is attributed after completing a scribble, and then during graphic production, the scheme representing meaning is not required; it is sufficient for the child to be able to produce a controlled scribbling, and subsequently use it as a cue for naming. Because the initial acquisition of language seems to require four sensorimotor schemes (Case, 1985), adding to these a pattern produced with controlled scribbling makes five schemes in all. Morra and Panesi (2017) reported evidence regarding the relation between the developmental growth of working memory capacity in toddlers and their progress from primitive to increasingly refined forms of scribbling and meaning attribution.

In another line of research, Riggs et al. (2013) suggested that the inhibition of immature forms of graphic activity (i.e., scribbling) is required in the transition to representational drawing. Following up that study, Simpson et al. (2017) provided evidence that, in preschoolers, fine motor control partly mediates the effect of inhibitory ability on drawing. Freeman and Adi-Japha (2008) also argued for a role of inhibitory control in drawing development; they suggested that suppressing earlier and less sophisticated “drawing rules” could be involved also in toddlers’ transition to representational drawing. In agreement with these studies, we hypothesize that toddlers also need to suppress a habitual drawing style, i.e., an early form of scribbling, to introduce a novel one for the production of more complex pictures such as diagrams or aggregates.

Also regarding language development, the evidence for a role of working memory and executive functions is extensive in preschoolers, but some studies on toddlers are also available. Gathercole (1995) found that, in 3-year olds, a measure of phonological working memory was related to their productive vocabulary, mean length of utterance, and variety of syntactic constructions that they produced in spontaneous speech. Newbury et al. (2016) found that 2-year-old’s working memory was the best predictor of their later development of both receptive and expressive language. Viterbori et al. (2012) found that, in 2-year olds, inhibitory control predicts expressive phonological accuracy, and inhibitory control and shifting jointly predict morphosyntactic skills. Cozzani et al. (2013) found that, in the 3rd year of life, the inhibition of prepotent responses was a strong predictor of syntactic ability in formulating sentences; moreover, they reported that a global measure of executive functioning and working memory, the Spin-the-Pots (Hughes and Ensor, 2005) predicted lexical, morphological, and syntactic abilities. Gandolfi and Viterbori (2020) found that interference suppression predicted language production ability in 2-year olds, as well as receptive morphosyntactic skills measured 1 year later.

The existence of sparse but consistent evidence of an influence of working memory and executive functions on the development of both language and drawing motivates us to investigate more systematically the developmental relations between these two representational systems, and their cognitive underpinnings. Panesi and Morra (under review) carried out a study with preschoolers (3–6-year-old) and found that, in that age range, the development of language and drawing was correlated; however, there was no direct link between them. Instead, the common variance of drawing and language was largely accounted for by executive functions (inhibition, updating, and shifting), which in turn were strongly dependent on the developmental growth of working memory capacity. However, the pattern of relations could be different in toddlerhood. In particular, in younger children, language could have a more direct effect on drawing, because of the developmental lag between the emergence of decontextualized language and representational drawing.

In this article, we investigate the pattern of relations between measures of working memory capacity, executive function, receptive and expressive language, and drawing in the 3rd year of life. To do so, we administered to a sample of toddlers a varied and relatively large battery of measures for each construct. In the domain of language, we assessed vocabulary and grammar in both comprehension and production. For drawing, we used the same set of tasks and measures used by Morra and Panesi (2017), which taps both the development of scribbling and the emerging representational competence. For executive processes, we used a number of working memory, inhibition, and shifting tasks suitable for toddlers and well recognized in the literature, except one that is new – the Memory Span Spin-the-Pots (MSSP; Morra et al., 2021), which we constructed modifying the original Spin-the-Pots (Hughes and Ensor, 2005) in a way that emphasizes its working memory component.

As a preliminary step, we needed to examine the relation between working memory and executive functions in this age range. Panesi and Morra (2020, under review) found, in preschoolers, two correlated but distinguishable factors, one loading tests of executive functions (inhibition, updating, and shifting), and the other loading working memory capacity measures. However, a re-analysis of those data in two separate groups of younger and older preschoolers suggests that the distinction between these two factors was evident only in the older group. This is consistent with the literature, in which a distinction between inhibition and working memory often emerges only after the age of five (e.g., Usai et al., 2014), whereas in younger children they compose a single factor (e.g., Wiebe et al., 2011); for a review, see Morra et al. (2018).

Therefore, in the data analysis, we first examined whether one or more factors represented best the structure of executive abilities in our participants. Second, we examined the relations between language and drawing measures. Finally, we turned to the main goal of this study – clarifying the pattern of relations between language, drawing, and their cognitive underpinnings in the 3rd year of life.

## Materials and Methods

### Participants

A total of 80 children, 25–37 months old, recruited in nurseries in a large metropolitan city and in a small town in Italy, took part in this study. We included only monolingual Italian toddlers with typical development (no formal diagnosis of disability, language impairment, or behavior disorder). Two children did not complete one of the tasks; for this reason, the data analyses were carried out on 78 participants (mean age = 31.1 months, SD = 3.0 months; 51 boys, 27 girls). Parents provided informed consent for participation.

### Materials, Procedure, and Measures

#### Drawing Tasks

We administered three tasks that assess different aspects of drawing skills, with the same materials and procedure used by Morra and Panesi (2017). A free drawing task provided the measures of children’s scribbling ability and the meaning they attribute to their graphic productions. Drawing completion tasks provided measures of children’s emerging representational competence. The human figure drawing was used to tap the structure and the richness of content of an early-emerging drawing scheme.

##### Free Drawing

The experimenter gave the child a white sheet and a pencil and invited the child to draw whatever he/she wanted. The experimenter praised often the child during his/her activity and, in case no meaning was declared by the child, eventually asked “What are you drawing?” When the child had finished, the experimenter asked “Can you tell me what you have drawn?” For this task, we created three scales for visual control, form, and meaning, respectively. Regarding visual control, we assessed in a dichotomous way (yes/no) three aspects of the child’s behavior, i.e., (a) gaze always or almost always directed to the sheet of paper while drawing, (b) drawing only on the sheet of paper without going out of its borders, and (c) varying the gestures or the quality of the marks while looking at the paper. One point was granted in the visual control scale for each of these features of the child’s behavior. Regarding form, we created a four-point scale: (0) uncontrolled scribbling, (1) controlled scribbling, (2) closed shapes or diagrams, and (3) combinations or aggregates of diagrams; in this scale, the transition from uncontrolled to controlled scribbling was considered as only a first step toward the depiction of better organized forms, which require increasingly controlled line production. We considered scribbling uncontrolled when the visual control score was 0 or 1, and controlled when the visual control score was 2 or 3 but the child did not draw closed shapes/diagrams or diagram combinations/aggregates (as defined by Kellogg, 1969). Regarding meaning, we created a three-point scale: (0) the child did not attribute any meanings verbally, either while drawing or after, (1) the child attributed some verbal meaning while drawing or after, and (2) coherent meaning, i.e., the child attributed the same meaning to the same graphic element on at least two occasions (before, while, or after drawing).

##### Drawing Completion

**Figure 1:**
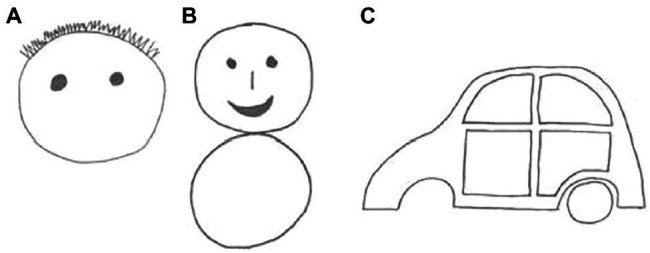
Stimuli used for the drawing completion task: **(A)** face, **(B)** human figure, and **(C)** car; **(A)** and **(B)** adapted from Freeman (1980); **(C)** adapted from Yamagata (2001).

Three incomplete pictures (a face, a human figure, and a car) were used in turn (see Figure 1); these stimuli were taken from previous studies (Freeman, 1980; Yamagata, 2001). The face was presented first, inviting the child to identify it. Then, the experimenter asked the child what was missing in it, and in case the child identified any missing element, encouraged him/her to draw it. The experimenter explicitly asked the child to draw the nose, and then to draw the mouth, in case the child did not identify spontaneously these missing elements. Finally, the experimenter asked the child if anything else was missing, and in case the child mentioned any relevant item (e.g., the ears or the hat), encouraged him/her to draw it. The same procedure was repeated for the human figure (where the elements explicitly required by the experimenter in case the child did not mention them were the arms, the legs, and the tummy button) and the car (where the explicitly required elements were a wheel and the doorknob). We constructed three scales to measure the child’s ability to (a) identify missing elements, (b) place the missing elements in appropriate positions, and (c) use appropriate shapes or marks to denote these elements. One point was scored, respectively, (a) for each element spontaneously identified, (b) for each element drawn in an appropriate position, and (c) for each element represented with appropriate marks (see Morra and Panesi, 2017 for further detail). Furthermore, we created a scale for diffuse scribbles in drawing completions, by counting the pictures on which the child made large scribbles in irrelevant positions.

##### Human Figure Drawing

The experimenter gave the child a white sheet and a pencil and asked him/her to draw a certain person (usually, the child’s mother or a favorite teacher). We created a three-point scale for its structure: (0) absence of structure, (1) tadpole figure or face only, and (2) differentiated head and trunk. Moreover, we scored in a dichotomous way (0/1) the presence of the following body parts: eyes, mouth, arms, legs, and other elements (e.g., nose, ears, and hair; i.e., various elements that appeared less frequently in the participants’ drawings were collapsed into a single “other” category). An overall scale for the human figure drawing was created by summing the points awarded for the structure and the body parts.

#### Language Tasks

We used a comprehensive test [Test del Primo Linguaggio (First Language Test; TPL)] that assesses both receptive and expressive language abilities in Italian (Axia, 1995). This test is standardized, with norms from 12 to 36 months. In this study, we administered the following four subscales.

##### Receptive Vocabulary

Twenty items were presented in a four-picture multiple-choice format; the child had to indicate the figure corresponding to the word produced by the experimenter. The score ranges from 0 to 20. In our sample, Cronbach’s alpha was .78.

##### Expressive Vocabulary

The child had to name 20 pictures showed one at a time by the experimenter. The score ranges from 0 to 20. In our sample, Cronbach’s alpha was .91.

##### Receptive Syntax

Twenty items were presented in a four-picture multiple-choice format; the child had to indicate the figure corresponding to the sentence produced by the experimenter. The score ranges from 0 to 20. In our sample, Cronbach’s alpha was .91.

##### Expressive Syntax

Twenty vignettes illustrating simple actions were presented. The child was required to describe each of them in turn. Each item was scored as follows, according to the test manual: 0 points when the child did not answer or gave an incorrect response; 1 point when the child responded with a single noun; 1.5 points when the child’s response was a verb; 2 points when the child gave a response composed by a subject and a verb; and 3 points when the child’s response was composed by more than two words. The score ranges from 0 to 60. In our sample, Cronbach’s alpha was .97.

#### Working Memory Tasks

Working memory capacity is defined as the number of chunks, schemes, or information units that a person can simultaneously attend to. A measure that is suitable for this age range is the Imitation Sorting Task (IST; Alp, 1994). Moreover, we used a modified version of Hughes and Ensor’s (2005) Spin-the-Pots, which we structured as a memory span task, and we called MSSP (Morra et al., 2021).

##### Imitation Sorting Task

The IST, according to Alp (1994), has a high test–retest reliability (*r* = 0.80); it is predictive of subsequent achievement in kindergarten (Fitzpatrick and Pagani, 2012) and has already been used in drawing research (Morra and Panesi, 2017). This task was presented as an imitation game; on each item, the experimenter sorted some objects into two canisters and then had the child imitate that sorting. The task is organized into eight levels of increasing difficulty (from one to eight objects per item). At level 1, there was only one item, requiring the child to place one toy into a canister. From level 2, the experimenter sorted the objects into two containers and the child was required to reproduce the demonstrated sorting. For each level from 2 to 8, there were five items. On each item, in case the child did not separate correctly the toys in the two canisters, the experimenter allowed him/her a second attempt. In case the child was successful on two of the first three items for level, the fourth and fifth items were skipped, and testing moved directly to the next level. A level was scored as passed in case the child correctly imitated the experimenter’s sorting in two of the first three items, or in three items out of five. Testing was discontinued when child failed two consecutive levels. The score indicates the highest level passed successfully (range: 0–8).

##### Memory Span Spin-the-Pots

This is a novel working memory task for young children, in which the child must retrieve objects placed under cups turned upside down. The main difference from the original Spin-the-Pots (Hughes and Ensor, 2005) is its span-like structure, with increasing numbers of hidden objects as the task proceeds. Moreover, this task has an “easy” and a “difficult” condition. In the “easy” condition, cups in different colors were arranged on a circular tray that remained static; the experimenter placed each target object under a cup, and after 3 s allowed the child to search for it. There were three levels, and at each level, there were three items. On level 1 items, the child had to find an object hidden in one of three pots; on level 2, the child had to find the objects hidden in two out of five pots; and on level 3, the child had to find the objects hidden in three out of seven colored pots. Testing was discontinued when child failed all the three items on a level. In the “difficult” condition, after placing the target objects under the cups, the experimenter covered the apparatus with a blanket, spun the tray by 90°, and uncovered it, allowing the child to search for the targets; all the rest was the same as in the easy condition. In each condition, one point was awarded for each correct item. The score ranges from 0 to 9 in each condition.

#### Inhibition Tasks

Inhibition is one of the three executive functions defined by Miyake et al. (2000). It is defined as the ability to refrain from prepotent responses and to resist interference from irrelevant information. We used three inhibition tasks extensively used in previous research with toddlers: the Circle Tracing task (adapted from Bachorowski and Newman, 1990), the Shape Stroop (Kochanska et al., 2000), and the Tower Building (Kochanska et al., 1996).

##### Circle Tracing

This task assesses the ability to control an ongoing motor response. We used a cardboard square on which a circle (8.5 cm in radius) was printed, with a small arrow indicating the starting point. In a first baseline condition, the child moved a doll around the circle, and in a second condition, the child was required to move as slow as he/she could a toy snail around the circle. Following this instruction requires inhibitory control. The score was calculated as the proportion of the slowdown to the total time in both conditions, using the following formula: (T2 − T1)/(T1 + T2), where T1 is the time recorded in the baseline condition and the T2 is the time recorded in the second inhibitory condition. Gandolfi and Viterbori (2020) reported an acceptable test–retest reliability (*r* = 0.57) in a sample of preschoolers.

##### Shape Stroop

This task assesses the ability to focus on a subdominant perceptual feature and to inhibit a prepotent response to a perceptually salient but misleading stimulus. In a preliminary phase, the experimenter showed the child colored individual drawings of large and small apples, oranges, and bananas. The experimenter named them in turn and asked the child to point to each. In the test phase, the experimenter showed the child three cards depicting a small fruit nested in a different large fruit (e.g., a small banana in a large orange) and asked the child to point to each of the small fruits. The score indicates the number of correct responses (range: 0–3). In our sample, Cronbach’s alpha was .62 (based on only three dichotomous items). However, a meta-analysis by Petersen et al. (2016) indicates that this task is useful as a measure of inhibitory control in the age range from 20 months to 3.5 years.

##### Tower Building

This task assesses the ability to take turns and to inhibit a prepotent response, as in go-no-go tasks. The child had to take turns with the experimenter in building together a tower, using 20 wooden blocks. The score indicates the number of correct turns (range: 0–10). Both Kochanska et al. (1996) and Gandolfi and Viterbori (2020) reported good or excellent reliability for this measure, and Petersen et al. (2016) concluded that it is useful in the age range from two to four and a half years.

#### Shifting Task

Another executive function is shifting. It is defined as the ability to switch smoothly between different tasks, rules, or cognitive operations. The Reverse Categorization (Carlson et al., 2004) is appropriate for this age.

##### Reverse Categorization

This task assesses the ability to classify objects according to different rules, switching from one to another. The experimenter showed the child two buckets and 12 blocks (six small and six larger). In a pre-switch phase, the experimenter asked the child to sort large blocks into the large bucket and small blocks into the small bucket. In a post-switch phase, the experimenter reversed these rules and asked the child to sort large blocks into the small bucket and small blocks into the large one. The score was the number of correct placements in the post-switch phase (range 0–12). In our sample, Cronbach’s alpha was .90.

#### General Procedure

Children took part in four individual sessions, carried out in the morning in a quiet room, and lasting approximately 20 min each. In the first session, the experimenter administered an inhibition task (Circle Tracing) and a working memory task (IST), in this order. Also in the second session, the child performed an inhibition task (Shape Stroop) and subsequently a working memory task (MSSP, easy condition first and difficult condition second). In the third session, all the drawing tasks were administered in the following order: free drawing, completion tasks, and human figure drawing; subsequently, in the same session, an inhibition task (Tower Building) was administered. In the fourth session, the TPL and a shifting task (Reverse Categorization) were administered, in this order.

## Results

### Preliminary Analyses

Descriptive statistics for all the tasks are reported in Table 1. Because of the skewness and kurtosis of two variables, for all further analyses, we submitted one of them to an exponential transformation (*e* power the proportion of correct responses in receptive vocabulary) and the other one, structure and body parts of the human figure drawing, to a square root transformation.

**Table 1:** Descriptive statistics.

Tasks and measures	Mean	Std. dev.	Min	Max	Skew	Kurtosis
***Free drawing***						
Visual control	2.71	0.51	1	3	−1.50	1.37
Form	1.97	0.66	0	3	−0.25	0.15
Meaning	1.00	0.84	0	2	0.00	1.58
***Drawing completions***						
Identifications	2.49	1.95	0	8	0.87	0.54
Correct placement	4.51	2.22	0	10	−0.09	−0.28
Appropriate marks	3.88	2.35	0	10	0.12	−0.55
Diffuse scribblings	0.95	1.12	0	3	0.79	−0.80
***Human figure drawing***						
Structure and body parts	0.62	1.45	0	6	2.45	5.19
***language test (TPL)***						
Receptive vocabulary	17.88	2.52	5	20	−2.66	9.61
Expressive vocabulary	12.81	5.09	0	20	−1.49	1.45
Receptive syntax	14.76	5.23	0	20	−1.80	2.43
Expressive syntax	29.08	19.16	0	58	−0.36	−1.30
***Working memory tasks***						
Imitation sorting task (IST)	3.46	1.63	1	8	1.31	1.39
MSSP, easy condition	3.86	1.69	0	8	−0.02	−0.49
MSSP, difficult condition	2.19	1.43	0	6	0.33	−0.21
***Inhibition tasks***						
Circle tracing	0.12	0.28	−0.50	0.85	−0.10	−0.39
Shape stroop	1.69	1.12	0	3	−0.21	−1.33
Tower building	3.77	3.36	0	10	0.51	−1.10
***Shifting task***						
Reverse categorization	9.09	3.51	0	12	−0.98	−0.11

Zero-order (Pearson) and partial correlations controlled for age between working memory capacity, inhibition, and shifting measures are reported in Table 2. All the measures correlated with age, except Circle Tracing and Shape Stroop. As often is the case in research on executive functioning in young children (e.g., Carlson et al., 2014), most correlations were rather low. The three working memory measures showed reasonable correlations with one another, all were significant. Among inhibition measures, the Shape Stroop correlated significantly with the Tower Building, but the Circle Tracing did not correlate significantly with them. The Tower Building correlated also with the IST, the easy condition of MSSP, and the Reverse Categorization. The Reverse Categorization correlated significantly also with the IST and the difficult condition of MSSP. However, only four correlations remained significant when age was partialled out. Having seven measures in this table, i.e., 21 correlations between pairs of measures, if they all were uncorrelated in the population, the chance expectation with a threshold of *p* = 0.05 would be to find approximately one significant correlation. The finding of nine correlations significant at *p* < 0.05 is highly significant (*p* < 0.001) at a binomial test, and also the finding of four partial correlations significant at *p* < 0.05 is significant (*p* < 0.02) at a binomial test.

**Table 2:** Pearson correlations and partial correlations between working memory, inhibition, and shifting measures.

	IST	MSSP easy	MSSP diffic.	Circle	Shape	Tower	Reverse
Age	0.50^**^	0.23^*^	0.38^**^	0.09	0.12	0.27^**^	0.34^**^
IST		0.24^*^	0.22^*^	0.15	0.15	0.24^**^	0.34^**^
MSSP easy	0.15		0.29^**^	0.09	−0.04	0.23^*^	0.00
MSSP difficult	0.03	0.23^*^		0.18	0.11	0.18	0.31^**^
Circle tracing	0.12	0.07	0.16		0.11	0.02	0.15
Shape stroop	0.11	−0.07	0.07	0.10		0.23^*^	0.14
Tower building	0.13	0.18	0.08	0.00	0.21^*^		0.22^*^
Reverse categorizat.	0.21^*^	−0.08	0.21^*^	0.13	0.10	0.14	

Zero-order Pearson correlations above diagonal; partial correlations (with age partialled out) below diagonal. IST, imitation sorting task; MSSP, memory span spin-the-pots. df = 76 for the zero-order correlations and df = 75 for the partial correlations.

* *p* < 0.05;

** *p* < 0.01.

To determine whether working memory capacity and executive function measures load on the same factor or two different factors, we ran first an exploratory factor analysis, with principal axes extraction. One factor accounted for 18.6% of the total variance. The factor loadings are reported in Table 3. We also tried a two-factor solution, but the extraction process did not converge even after 1,000 iterations.

**Table 3:** Factor loadings in the exploratory factor analysis of working memory capacity, inhibition, and shifting measures.

Tasks	Loadings
Imitation sorting task	0.56
MSSP, easy condition	0.34
MSSP, difficult condition	0.52
Circle tracing	0.27
Shape stroop	0.27
Tower building	0.44
Reverse categorization	0.51

Extraction method: principal axis.

Subsequently, we ran a confirmatory factor analysis to test two theoretically different models: a one-factor model and a two-factor model. In the latter, we assumed that IST, MSSP easy condition, and MSSP difficult condition are working memory measures; and Circle Tracing, Shape Stroop, Tower Building, and Reverse Categorization are executive function measures. This would be a structure similar to the one found in preschoolers (3–6-year olds) by Panesi and Morra (2020). The fit indices for these models are presented in Table 4.

**Table 4:** Goodness of fit indices for the alternative measurement models (confirmatory factor analyses) of working memory capacity, inhibition, and shifting measures.

Model	*χ*^2^	df	*p*	GFI	CFI	RMSEA	SRMR	AIC
**One-factor**	**12.20**	**14**	**0.59**	**0.96**	**1.00**	**0.000**	**0.062**	**40.20**
Two-factors	12.18	13	0.51	0.96	0.98	0.000	0.062	42.18

**Figure 2:**
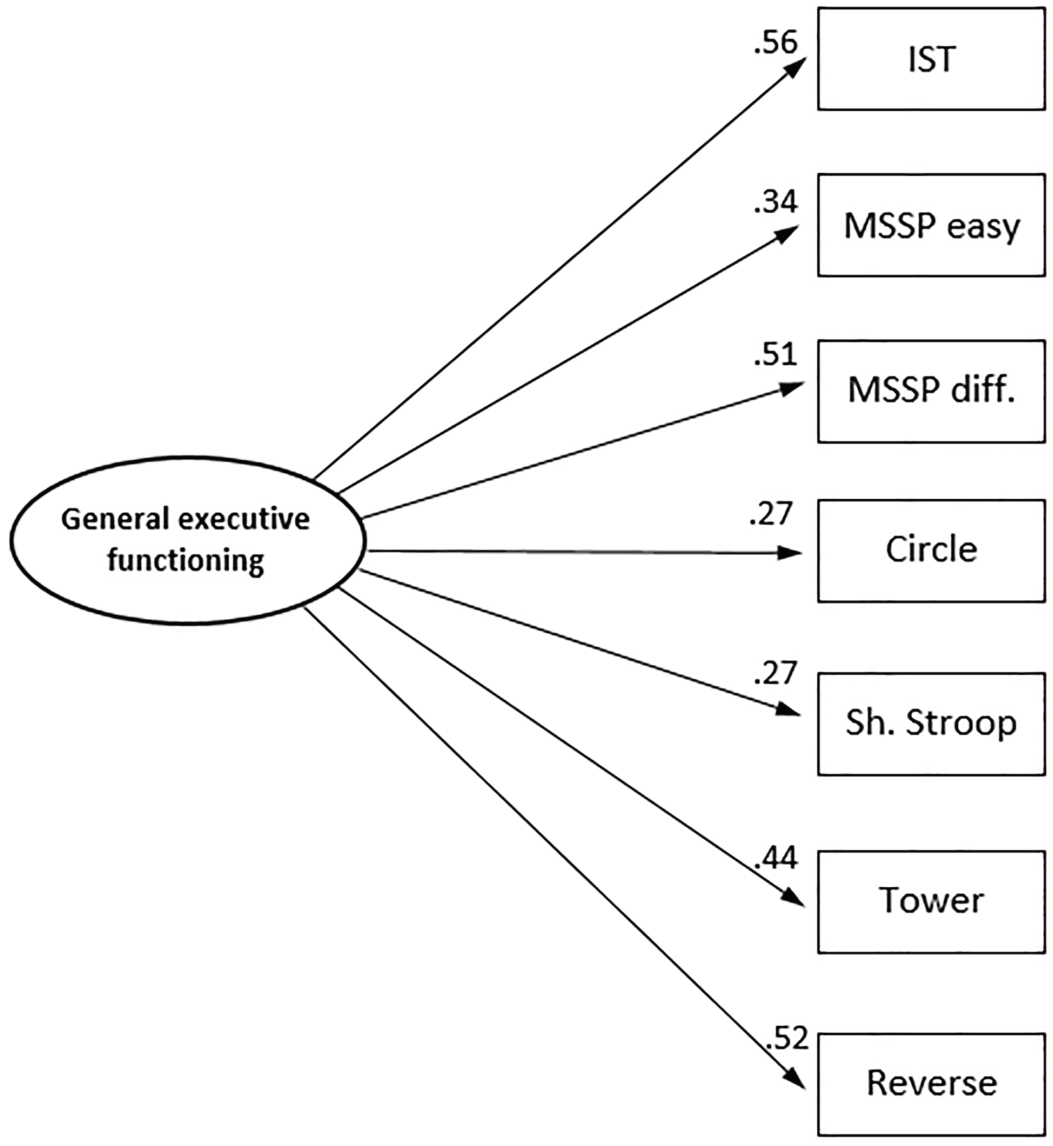
Best-fitting measurement model (one-factor model) for the working memory capacity, inhibition, and shifting measures. IST, imitation sorting task; MSSP easy, memory span spin-the-pots, easy condition; MSSP diff., memory span spin-the-pots, difficult condition; Circle, circle tracing; Sh. Stroop, shape stroop; Tower, tower building; Reverse, reverse categorization.

Endorsed model in bold; see Figure 2.

The fit indices for both models were generally good; some of them (CFI, AIC, and probability of the *χ*^2^) were slightly better for the one-factor model than the two-factor model. More important, it was possible to compare directly the two models by means of the *χ*^2^ difference, because merging two factors is mathematically equivalent to setting at the 1 correlation between those two factors, and therefore the models are nested.^2^ This test showed that the two-factor model did not fit the data better than the one-factor model (Δ*χ*^2^ = 0.02, df = 1, n.s.). This implies that the one-factor solution is more parsimonious, without any loss of relevant information. Moreover, in the two-factor model, the *phi* parameter representing the correlation between the two factors was 0.96; in other words, both latent variables nearly coincided.

In sum, all the results from both exploratory and confirmatory factor analyses consistently indicated that a single factor accounted best for all common variance between working memory capacity, inhibition, and shifting measures in this study. This is consistent with previous research on very young children, and different from the results with older preschoolers reported by Panesi and Morra (2020), where working memory capacity measures could be distinguished from an executive function factor that included inhibition, shifting, and updating measures. Consequently, in the following of this article, we shall use a measurement model for our measures of working memory capacity, inhibition, and shifting that includes a single factor, henceforth called general executive functioning. Figure 2 represents the relevant parameters in this model.

### Drawing and Language

Zero-order (Pearson) and partial correlations controlled for age between language and drawing measures are reported in Table 5. All measures correlated with age, except the Form and Meaning scales in the free drawing task. Age correlated most highly with the four measures derived from drawing completions and the four language measures (the correlation with diffuse scribbles was negative, as one could expect, because scribbling diffusely is an immature response in a drawing completion task).

**Table 5:** Pearson correlations and partial correlations between drawing and language measures.

	1	2	3	4	5	6	7	8	9	10	11	12
Age	0.19^*^	0.03	0.12	0.41^**^	0.57^**^	0.44^**^	−0.34^**^	0.26^*^	0.44^**^	0.37^**^	0.39^**^	0.50^**^
1 – Visual control		0.36^**^	0.12	0.17	0.25^*^	0.36^**^	−0.46^**^	0.10	0.15	−0.02	0.26^*^	0.12
2 – Form scale	0.36^**^		0.05	0.24^*^	0.24^*^	0.27^**^	−0.26^**^	0.34^**^	0.10	0.07	0.20^*^	0.18
3 – Meaning scale	0.10	0.04		0.29^**^	0.18	0.19	−0.13	0.13	0.21^*^	0.31^**^	0.14	0.31^**^
4 – Identifications	0.10	0.25^*^	0.27^**^		0.57^**^	0.57^**^	−0.20^*^	0.28^**^	0.38^**^	0.35^**^	0.32^**^	0.48^**^
5 – Corr. placem.	0.17	0.27^**^	0.14	0.45^**^		0.83^**^	−0.62^**^	0.28^**^	0.42^**^	0.27^**^	0.51^**^	0.46^**^
6 – Appr. marks	0.31^**^	0.29^**^	0.15	0.48^**^	0.70^**^		−0.55^**^	0.36^**^	0.34^**^	0.27^**^	0.46^**^	0.46^**^
7 – Diffuse scrib.	−0.43^**^	−0.27^**^	−0.09	−0.07	−0.55^**^	−0.48^**^		−0.24^*^	−0.31^**^	−0.18	−0.39^**^	−0.35^**^
8 – Human figure	0.05	0.35^**^	0.10	0.19^*^	0.17	0.28^**^	−0.17		0.21^*^	0.25^*^	0.25^*^	0.38^**^
9 – Recept. voc.	0.07	0.10	0.17	0.24^*^	0.23^*^	0.19	−0.19^*^	0.11		0.53^**^	0.52^**^	0.49^**^
10 – Expr. voc.	−0.10	0.07	0.29^**^	0.24^*^	0.08	0.12	−0.06	0.17	0.44^**^		0.36^**^	0.70^**^
11 – Recept. synt.	0.20^*^	0.21^*^	0.10	0.19	0.38^**^	0.35^**^	−0.29^**^	0.17	0.42^**^	0.26^*^		0.43^**^
12 – Expr. synt.	0.03	0.20^*^	0.29^**^	0.35^**^	0.25^*^	0.30^**^	−0.22^*^	0.30^**^	0.35^**^	0.64^**^	0.29^**^	

Zero-order Pearson correlations above diagonal; partial correlations (with age partialled out) below diagonal. Corr. Placem., correct placements; Appr., appropriate; Scrib, scribbles; Recept., receptive; Expr., expressive; Voc., vocabulary; Synt, syntax. df = 76 for the zero-order correlations and df = 75 for the partial correlations.

* *p* < 0.05;

** *p* < 0.01.

All six correlations between language measures were significant and moderate or high (from 0.36 to 0.70), and all of them remained significant with age partialled out.

Considering in Table 5 the correlations between drawing measures, the visual control scale correlated with the form scale, but this correlation is partly artefactual, because achieving visual control is a part of the form scale. The meaning scale, instead, did not correlate with the other two measures obtained from the free drawing task. The form scale correlated with all drawing completion measures and with the human figure, the visual control scale correlated with all drawing completion measures except identifications, and the meaning scale correlated with only one drawing measure, i.e., identifications. All measures taken in the drawing completion task correlated with one another, and all of them correlated with the human figure. In all, 20 out of 28 correlations between drawing measures were significant, and 16 of them remained significant with age partialled out. Notably, also with age partialled out did the form scale correlate (in most cases at *p* < 0.01) with the human figure and with all measures in the drawing completion task, and the human figure correlated not only with the form scale (*p* < 0.01), but also with appropriate marks (*p* < 0.01) and identifications (*p* < 0.05) in the drawing completion.

For the purpose of this article, among the correlations reported in Table 5, the most relevant are those between a language and a drawing measure. There were four language measures and eight drawing measures, which makes a total of 32 correlations. With 32 correlations and a threshold of *p* = 0.05, the chance expectation would be to find 1.6 significant correlations (i.e., one or two). We found that 24 correlations out of 32 were significant at *p* < 0.05; this outcome is highly significant (*p* < 0.001) at a binomial test. Moreover, 17 of these correlations remained significant with age partialled out, which also is significant (*p* < 0.001) at a binomial test. In particular, the syntax measures showed the highest correlations with drawing measures. With age partialled out, the highest correlations between a language and a drawing measure involved receptive syntax with correct placements (*r* = 0.38) and appropriate marks (*r* = 0.35), and expressive syntax with identifications (*r* = 0.35), appropriate marks (*r* = 0.30), and human figure (*r* = 0.30, all *p* < 0.01). This seems consistent with the analogies between language syntax and drawing suggested by Willats (1985) and Cohn (2012). One can also note in Table 5 that, also with age partialled out, each of the eight drawing measures correlated significantly with at least one language measure – often, with more than one. This suggests that language and drawing development are intertwined in toddlers.

To clarify the relation between drawing and language and to determine whether they can be considered two different and interconnected representational systems, we carried out exploratory and confirmatory factor analyses. In these analyses, we did not include the visual control scale to avoid possible artifacts due to its partial overlap with the form scale. Thus, seven drawing measures and four language measures were analyzed.

We ran an exploratory factor analysis with principal axis extraction and oblimin rotation, which allows for correlated factors. Two factors emerged, which accounted for 46.6% of the total variance. The correlation between factors was 0.53. The factor loadings (pattern matrix) and the correlations of variables with factors (structure matrix) are reported in Table 6. Most drawing measures loaded on the first factor (except the meaning scale) and most language measures (except receptive syntax) loaded on the second. However, the structure matrix shows that most variables actually correlated with both factors, again suggesting that language and drawing are intertwined at this age.

**Table 6:** Factor loadings and structure matrix in the exploratory factor analysis of language and drawing measures.

	Pattern matrix (factor loadings)		*Structure matrix (correlations)*	
	Factor 1	Factor 2	*Factor 1*	*Factor 2*
Form scale	0.36		*0.35*	
Meaning scale		0.35		*0.37*
Identifications	0.43		*0.58*	*0.51*
Correct placements	0.94		*0.92*	*0.46*
Appropriate marks	0.91		*0.89*	*0.44*
Diffuse scribblings	−0.65		*−0.63*	*−0.32*
Human figure	0.30		*0.40*	*0.35*
Receptive vocabulary		0.54	*0.46*	*0.63*
Expressive vocabulary		0.97	*0.31*	*0.86*
Receptive syntax	0.40		*0.56*	*0.51*
Expressive syntax		0.74	*0.54*	*0.82*

Extraction method: principal axis. Rotation method: oblimin. Coefficients below 0.3 in absolute value omitted.

To test whether drawing and language were best represented by one or two factors, we ran a confirmatory factor analysis. We started by creating a one-factor model (Model A), loading all language and drawing measures, and a two-factor model (Model B) in which language was distinguished from drawing. Receptive vocabulary, expressive vocabulary, receptive syntax, expressive syntax were posited to load on one factor, whereas form, meaning, identifications, correct placements, appropriate marks, diffuse scribbles, and human figure were posited to load on the other factor. As shown in Table 7, Model A was clearly inadequate. Its *χ*^2^ was highly significant; according to the conventional thresholds for model evaluation (e.g., Schermelleh-Engel et al., 2003), GFI and CFI were too low, RMSEA was excessive, and only SRMR was barely acceptable. Model B fit the data better than Model A; their *χ*^2^ difference was highly significant (Δ*χ*^2^ = 56.58, df = 1, *p* < 0.001), and all indices improved when two factors were posited. However, Table 7 also shows that Model B did not fit the data perfectly; its CFI and SRMR were acceptable, but the other indices were not acceptable, and the *χ*^2^ was still significant. Examining the modification indices, we noted that three *theta-delta* parameters (i.e., covariances between errors of observed variables) should be included in the model. Thus, we created Model C, by freeing in Model B the three *theta-delta* parameters representing the error covariances between (a) expressive vocabulary and expressive syntax, (b) correct placements and appropriate marks, and (c) identifications and diffuse scribbles.^3^ Model C fit the data better than Model B; their *χ*^2^ difference was highly significant (Δ*χ*^2^ = 29.65, df = 3, *p* < 0.001), and all indices improved when these three error covariances were added. Model C fit the data very well; CFI and RMSEA were excellent, and also the other indices were fully acceptable. Moreover, the *χ*^2^ value was nonsignificant, and the *χ*^2^/df ratio was smaller than 1.

**Table 7:** Goodness of fit indices for the alternative measurement models (confirmatory factor analyses) of language and drawing measures.

Model	*χ*^2^	df	*p*	GFI	CFI	RMSEA	SRMR	AIC
A *(one-factor)*	124.99	44	<0.001	0.77	0.88	0.15	0.10	168.99
B *(two-factors)*	68.41	43	<0.01	0.86	0.96	0.088	0.09	114.41
**C *(two****-**factors and error covar.)***	**38.76**	**40**	**=0.053**	**0.92**	**1.00**	**0.000**	**0.065**	**90.76**

**Figure 3:**
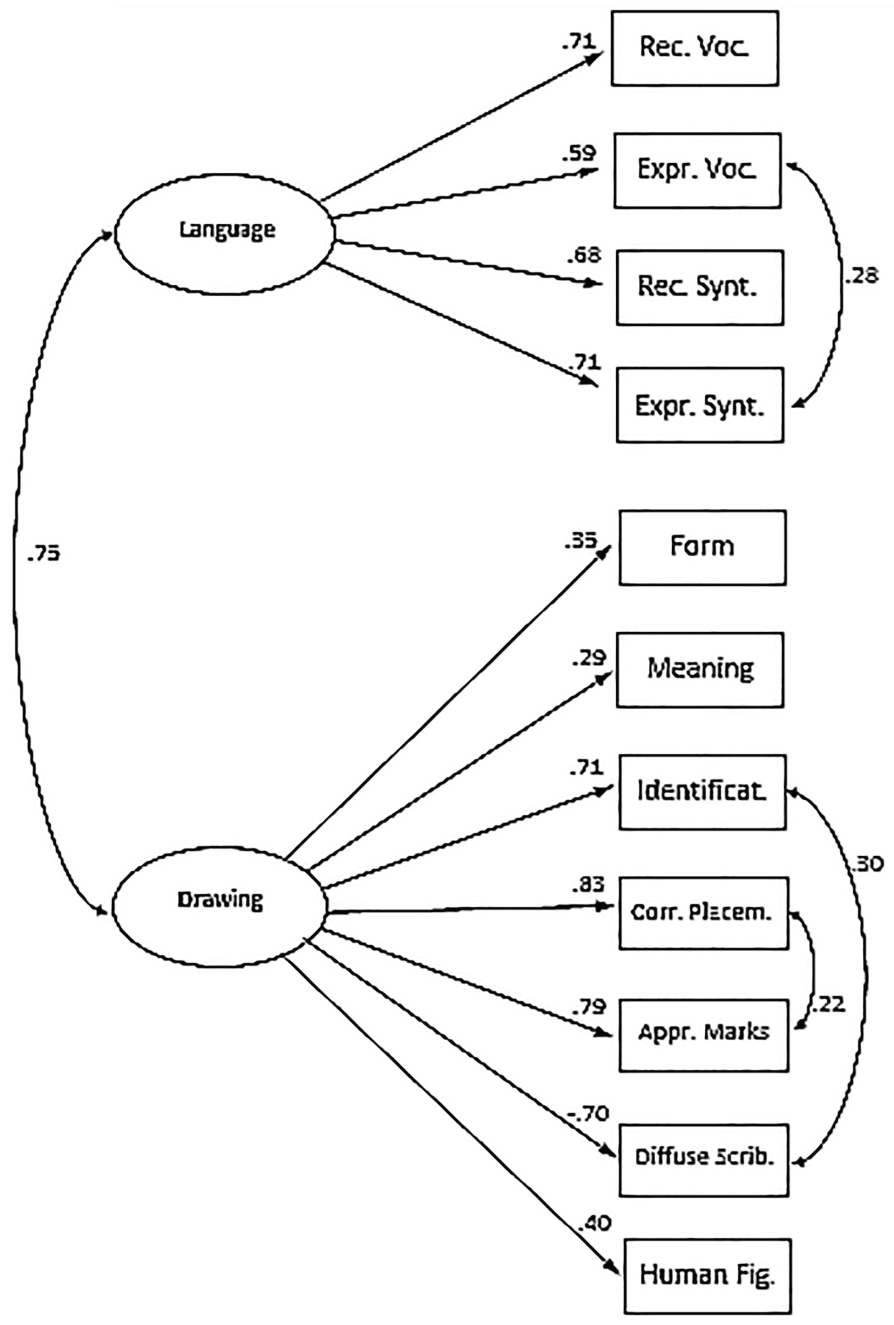
Best-fitting measurement model (two-factor model with error covariances) for the language and drawing measures. Rec., receptive; Expr., expressive; Voc., vocabulary; Synt., syntax; Identificat., identifications; Corr. Placem., correct placements; Appr., appropriate; Scrib., scribbles; Fig., figure.

Endorsed model in bold; see Figure 3.

The best-fitting model is presented in Figure 3. One can note that, in this model, the correlation between the drawing and language latent variables was rather high, i.e., *phi* = 0.75. To some extent, this value could be an overestimation, because for the sake of simplicity each measure was posited to load on only one factor, and many factor loadings were set at zero (e.g., see Asparouhov and Muthén, 2009). However, even discounting possible overestimation, the finding of a rather high correlation between the two latent variables is consistent with the numerous significant correlations between a language and a drawing measure.

### Executive Control, Language, and Drawing

Table 8 presents the correlations between a language or a drawing measure, and one of the measures of working memory, inhibition, and shifting. The correlations were generally not high, but still, they indicated a sizable contribution of these domain-general cognitive functions to both language and drawing.

**Table 8:** Pearson correlations between a language or a drawing measure, and one of the working memory, inhibition, and shifting measures.

	IST	MSSP easy	MSSP diffic.	Circle	Shape stroop	Tower build.	Reverse categ.
Visual control	0.23^*^	0.06	0.04	0.09	0.04	0.26^*^	0.08
Form scale	0.04	0.01	−0.08	0.10	0.20^*^	0.31^**^	0.16
Meaning scale	0.07	−0.04	0.01	0.17	0.21^*^	0.07	0.10
Identifications	0.14	0.20^*^	0.12	0.19	0.08	0.29^**^	0.21^*^
Correct placements	0.37^**^	0.04	0.23^*^	0.08	0.04	0.20^*^	0.40^**^
Appropriate marks	0.25^*^	−0.01	0.17	0.01	0.12	0.20^*^	0.32^**^
Diffuse scribbles	−0.30^**^	−0.12	−0.16	−0.09	0.02	−0.21^*^	−0.35^**^
Human figure	0.05	0.08	0.19^*^	0.20^*^	0.19^*^	0.21^*^	0.25^*^
Receptive vocabulary	0.20^*^	0.14	0.31^**^	0.09	0.16	0.09	0.37^**^
Expressive vocabulary	0.10	0.28^**^	0.15	0.22^*^	0.06	0.14	0.36^**^
Receptive syntax	0.21^*^	0.16	0.22^*^	0.18	0.11	0.29^**^	0.42^**^
Expressive syntax	0.28^**^	0.21^*^	0.32^**^	0.33^**^	0.18	0.17	0.53^**^

Drawing measures in the upper part, and language measures in the lower part of the table. Diffic., difficult; Build., building; Categ., categorization. df = 76 for the zero-order correlations and df = 75 for the partial correlations.

* *p* < 0.05;

** *p* < 0.01.

Considering the correlations between a language measure and one of the measures of general executive functioning, 15 out of 28 correlations were significant, and eight of them remained significant with age partialled out. The chance expectation would be 1.4 significant correlations; both findings of 15 significant correlations and eight significant partial correlations are more than expected by chance (*p* < 0.001 in both cases). The Reverse Categorization was the executive function task that showed the highest correlations in this domain; it correlated significantly with all four language measures. The IST and the difficult condition of the MSSP correlated with three language measures each. With age partialled out, Reverse Categorization correlated significantly with Expressive Syntax (*r* = 0.45, *p* < 0.01), Receptive Syntax (*r* = 0.33, *p* < 0.01), Expressive Vocabulary (*r* = 0.27, *p* < 0.01), and Receptive Vocabulary (*r* = 0.26, *p* < 0.05); Circle Tracing correlated significantly with Expressive Syntax (*r* = 0.33, *p* < 0.01) and Expressive Vocabulary (*r* = 0.20, *p* < 0.05); the Tower Building correlated significantly with Receptive Syntax (*r* = 0.21, *p* < 0.05); and the easy condition of the MSSP correlated significantly with Receptive Vocabulary (*r* = 0.21, *p* < 0.01).

Considering the correlations between a drawing measure and one of the measures of general executive functioning, 23 out of 56 correlations were significant, and eight of them remained significant with age partialled out. Each drawing measure correlated with at least one executive task, and vice versa. The chance expectation would be 2.8 significant correlations; both findings of 23 significant correlations and eight significant partial correlations are higher than expected by chance (*p* < 0.001 and *p* < 0.01, respectively). The highest correlations were those between Reverse Categorization and Correct Placements (*r* = 0.40, *p* < 0.01), IST and Correct Placements (*r* = 0.37, *p* < 0.01), and Reverse Categorization and Diffuse Scribbles (*r* = −0.35, *p* < 0.01). The Tower Building correlated significantly with seven drawing measures, the Reverse Categorization with six, the IST and the Shape Stroop with three. With age partialled out, the Tower Building correlated significantly with the Form scale (*r* = 0.32, *p* < 0.01), Visual Control (*r* = 0.22, *p* < 0.05), and Identifications (*r* = 0.21, *p* < 0.05); Reverse Categorization correlated significantly with Correct Placements (*r* = 0.27, *p* < 0.01), Diffuse Scribblings (*r* = −0.27, *p* < 0.01), and Appropriate Marks (*r* = 0.21, *p* < 0.05); and the Shape Stroop correlated significantly with the Form scale (*r* = 0.20, *p* < 0.05) and the Meaning scale (*r* = 0.20, *p* < 0.05).

Having examined separately the correlations between each pair of tasks and having also tested the measurement models for language, drawing, and general executive functioning; now, we can turn to the “big picture” of the pattern of relations among these different facets of cognitive development. We shaped and tested structural equation models to examine the developmental associations among drawing, language, and general executive functioning. In particular, we investigated whether and to which extent, in toddlers, general executive functioning accounts for the development of drawing and language and contributes to the association between them. Note that, contrary to the measurement models (which only include correlations between latent variables), causal models posit *directional* causal effects among latent variables. Causal assumptions are a core feature of the structural equation modeling methodology. Assumptions on causal relations are made on the basis of prior research, theoretical knowledge, scientific judgment, or other plausible justifications, and then tested against the data (Bollen and Pearl, 2013). Moreover, it is advisable to compare different models that include different patterns of structural relations, i.e., different pathways of causal effects and assess which of the hypothesized causal patterns fits the data best, and therefore is most credible.

Examining the relations among the constructs of our interest using, in all, 19 observed variables would require a much larger sample. However, for practical reasons (in particular, the restrictions due to the covid-19 pandemic), now it would be impossible to collect more data. Therefore, we decided to use a reduced set of only seven variables, selected on the basis of the measurement models presented so far (see Figures 2, 3, and related text) to represent the intended latent constructs. For general executive functioning, we selected the working memory task with highest loading in the CFA (the IST), the inhibition task with highest loading (the Tower Building), and the only shifting task we used (Reverse Categorization), which also had a rather high loading in this prior analyses. For language, we selected Receptive Vocabulary and Expressive Syntax, so that both vocabulary and syntax are covered, and there are one receptive and one expressive language measures, and both measures loaded highly on the language factor in the CFA. For drawing, we selected one measure from the drawing completions (Appropriate Marks) and one from the “unscaffolded” drawing tasks (Human Figure); although the latter had a lower loading than most variables from drawing completions, we felt that choosing two measures from completions would be biased, and in the CFA the human figure was the measure with highest loading apart from those obtained from drawing completions.

To establish the best model, we proceeded in steps. First, as a start point, we created a model in which age (expressed in months) was the only exogenous (i.e., independent) variable, measured without error; and language, drawing, and general executive functioning were three endogenous (i.e., dependent) latent variables. Three *gamma* parameters from age to each endogenous variable and no *beta* parameter were included in this model. In other words, this model posited that age has causal effects on language, drawing, and executive functioning, but no effects were posited among these latent variables. This very simple initial model is referred in Table 9 as Model A. The estimated effects in this model were substantial, being 0.67, 0.56, and 0.71 for the regression on age of language, drawing, and general executive functioning, respectively. However, this model did not fit the data well; as shown in Table 9, GFI and CFI were a bit low, RMSEA and SRMR were excessive, and there was a significant misfit indicated by *χ*^2^ = 37.00 (df = 18), *p* < 0.01.

**Table 9:** Goodness of fit indices for the alternative models of structural relations among language, drawing, general executive functioning, and age.

Model	*χ*^2^	df	*p*	GFI	CFI	RMSEA	SRMR	AIC
A *(only relations with age)*	37.00	18	<0.01	0.89	0.92	0.12	0.11	73.00
B *(two beta parameters added)*	19.01	16	>0.26	0.94	0.98	0.049	0.054	59.01
**C *(two nonsignificant gamma parameters dropped)***	**19.03**	**18**	**>0.38**	**0.94**	**0.99**	**0.027**	**0.056**	**55.03**
D *(language affects executive function)*	20.91	18	>0.28	0.94	0.99	0.046	0.056	56.91

**Figure 4:**
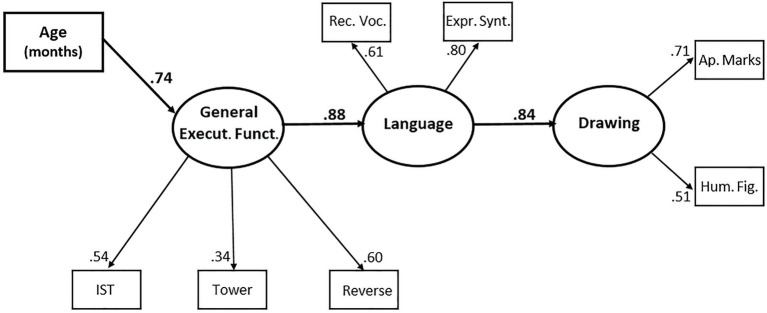
Best-fitting structural equations model for general executive functioning, language, and drawing. Execut. Funct., executive functioning; IST, imitation sorting task; Tower, tower building; Reverse, reverse categorization; Rec. Voc., receptive vocabulary; Expr. Synt., expressive syntax; Ap. Marks, appropriate marks; Hum. Fig., human figure.

Endorsed model in bold; see Figure 4.

The next step was including in the model causal effects between latent variables. On the basis of current knowledge and previous research reviewed in the section “Introduction,” it seems plausible to assume that language might affect drawing, and that executive functioning might affect both drawing and language. In fact, the modification indices for Model A suggested that we should include a relation between drawing and language and one between language and general executive functioning. A relation between drawing and general executive functioning, instead, was not included because it had a low modification index (i.e., it would not be significant). Therefore, we freed in the model two parameters, one for the regression of language on general executive functioning, and another for the regression of drawing on language (as mentioned above, the direction of these effects was specified according to theoretical expectations; however, see below for a test of an alternative causal pattern). In Table 9, this model is referred to as Model B. Including in the model these two structural relations, i.e., these causal effects, caused a notable improvement of the model’s fit, with a highly significant *χ*^2^ difference (Δ*χ*^2^ = 17.99, df = 2, *p* < 0.001). Table 9 also shows that all indices were good or acceptable, and better for Model B than Model A. However, in Model B, the *gamma* parameters for the effects of age on language and drawing were no longer significant.

Therefore, we tested Model C, which differed from Model B only because these two *gamma* parameters were fixed at zero. In other words, it was posited that the effect of age on language was not direct, but mediated by general executive functioning; and that also the effect of age on drawing was not direct, but mediated by the other latent variables. Removing these free parameters did not cause any significant loss of relevant information (Δ*χ*^2^ = 0.02, df = 2, n.s.), it rendered the model more parsimonious, and actually, some indices improved; in particular, the RMSEA and the CFI indicated an excellent fit. The *χ*^2^/df ratio was 1.06, which is another indication of good fit, and the test of close fit (i.e., the probability that RMSEA < 0.05) yielded *p* = 0.60. Therefore, we accept this model, which is represented in Figure 4.

In this model, there is a cascade of effects: age affects general executive functioning, which affects language, which in turn affects drawing. The regression parameters for each of these effects were rather high and highly significant (0.74, *p* < 0.001; 0.88, *p* < 0.001; and 0.84, *p* < 0.001, respectively).

As a possible alternative, we also tested a model in which the direction of the causal effect between language and general executive function was reversed. We did so in view of a debate in the literature, regarding the direction of this relation (e.g., Bishop et al., 2014; Slot and von Suchodoletz, 2018).^4^ Model D was conceptually similar to Model C, except that one *beta* parameter represented the regression of general executive functioning on language instead of vice versa, and the one *gamma* parameter represented the regression of language on age. Models C and D cannot be compared directly by means of a *χ*^2^ difference, because neither of them is embedded in the other; it is only possible to compare them indirectly, by examining their goodness-of-fit indices. As shown in Table 9, also the indices of Model D were acceptable, but the RMSEA and AIC were clearly worse for Model D than for model C. Consequently, we discarded Model D and concluded that Model C was the best fitting one, i.e., the most plausible.

## General Discussion

The results of this study can be summarized as follows. First, all the measures of working memory capacity, inhibition, and shifting loaded on a single factor (that we labeled general executive functioning). Second, language and drawing measures grouped in two distinguishable factors, which correlated substantially with each other. Third, the best fitting model assumed an underlying pattern of causal relations, in which the development of executive functioning had a considerable impact on language, which in turn affected considerably drawing skills.

The finding of a single factor for all executive functioning is consistent with previous research with 4-year olds or younger children – at least so far as “cool” executive functions or “conflict tasks” are concerned (Wiebe et al., 2011; Carlson et al., 2014; Mulder et al., 2014). There is some evidence in the literature (Carlson et al., 2014; Mulder et al., 2014) that “hot” executive functions or “delay tasks” can behave differently, but in our study we did not use these types of task.

The finding of substantial correlations between language and drawing, at the levels of both latent variables and pairs of single measures, rules out the view that verbal and visual representations are processed differently and along separate channels (e.g., Paivio, 1971), insofar as toddlers’ language and drawing are concerned. On the contrary, our final model of structural relations, shown in Figure 4, supports (at least in this age range) the view that language influences drawing (Freeman, 1972; Golomb, 1992; Callaghan, 2000; Toomela, 2002). The specific finding that syntax measures correlated highest with drawing measures seems consistent also with the idea of an analogy between language syntax and the “syntax” of drawing (Willats, 1985; Cohn, 2012).

An extreme interpretation of a single and unitary symbolic function is not supported by our results, because we found language and drawing to be distinct latent variables, albeit correlated. However, it is still possible to conceive the influence of language on drawing as mediated or embedded within a more general symbolic function. Our results are silent on this point, because drawing emerges developmentally later than language; therefore, we cannot know whether the influence of language on drawing, asserted in our final model, actually comes from language alone, or (also) from a more general symbolic function. To clarify this point, a similar study would be needed, including also some other, early-appearing symbolic representation – for instance, symbolic play, using standardized procedures for its assessment (e.g., Lewis et al., 2000). If it turned out that not only language, but also symbolic play affects drawing, that would be evidence for a role of a more general symbolic function.

The most relevant finding of this study is the support for a hypothesized pattern of relations among executive function, language, and drawing in the third year of life. This finding sheds some light on the developmental relations between different representational domains, and among different processes in the developing mind. It clearly has theoretical implications, because it emphasizes a major role of general executive processes in cognition, already in 2-year olds. Furthermore, it contributes to the criticism of modular views of cognition and cognitive development. Not only do we need to criticize the modular theories of human mind for their presuppositions on innateness (Bates et al., 1996); indeed, our findings suggest that modular theories can be criticized also for their claim that modules are encapsulated. They are not – given the massive contribution of domain-general executive processes to the developing language and the important connection between toddlers’ language and their drawing skills. What emerges from our results is the picture of a strongly interconnected developing mind.

It should be noted that this study replicated only in part the results of Morra and Panesi (2017). In that study, we used only drawing measures and one working memory task (the IST) and found highly significant correlations between working memory and drawing. In the current study, we replicated the finding of a relation between drawing and general executive functioning, albeit mediated by language. However, not all the correlations between the IST and the drawing measures were significant, and they were generally lower than those reported by Morra and Panesi (2017). It is unclear whether these differences were due merely to random variation between samples, or some minor methodological differences between these studies had some impact on the size of the correlations. Unfortunately, the relations between working memory development and the emergence of drawing are still under-researched; further replications and control of methodological details are needed to clarify this point.

As discussed above, in this study, we found a cascade of effects – age on general executive functioning, on language, and on drawing. It is important to stress that this pattern could be characteristic of toddlerhood, and need not remain the same in subsequent developmental phases. In fact, we carried out a similar study with preschoolers, 3–6 years old (Panesi and Morra, under review). In preschoolers, we found direct effects of executive functions on both drawing and language, and the correlation between language and drawing was fully accounted for by the dependence of both of them on executive functions. Comparing and combining the results of both studies, we think that they jointly provide important information on the course of drawing development. As widely known, drawing starts as “scribbling,” i.e., as a sensorimotor activity that is void of representational meaning. During the 3rd year, toddlers improve their graphic skills for the production of more refined forms, and – even more important – they also get to understand that drawings can have a representational meaning, as evident especially in the drawing completion tasks, in this study as well as in Morra and Panesi (2017). This study indicates that the transition from purely sensorimotor scribbling to representational drawing is driven by the child’s mastering of language. However, it also seems that, once the major leap from scribbling to representation is made (perhaps in the 4th year of life), drawing becomes more independent of language, and their parallel development is mainly a consequence of the importance of executive functions for both of them (Panesi and Morra, under review).

This study has some practical implications. First, given this demonstration of importance of executive functioning in cognitive development, it would be useful to foster it. Educators in nurseries could involve toddlers in games that enable them to practice executive control. For instance, Traverso et al. (2019) described an executive function training for preschoolers that had also far transfer effects, and Scionti et al. (2020) provided a meta-analysis of related training studies; similar training programs could be devised also for younger children. Also, the results of Morra and Panesi (2017) suggested that drawing completion can prompt better performance on a subsequent human figure drawing; educational activities based on dialog on incomplete drawings and completion games could perhaps train the toddlers’ executive functions in the context of verbal communication and pictorial representations. These hypothetical suggestions could be tested in future studies. Furthermore, considering the impact of language on the transition from scribbling to drawing, it would be interesting to investigate whether preschoolers with specific language impairment are also delayed in representational drawing.

The ambitious goal of this study, and a related one on preschoolers, was to investigate the relations between two representational systems, and the role of working memory and executive functions in their development. The results are promising, and we think that this approach could be extended further, including also other representational systems and representational domains. We have already discussed the reasons for including also symbolic play in future studies. Another relevant domain would be numerical cognition (e.g., Le Corre and Carey, 2007), because language seems to be involved in the acquisition of numerical concepts in early childhood, and in older children there is extensive evidence for a role of inhibition and working memory in math cognition. Extending the approach of this study to other domains would enable researchers to map the connections among different aspects of cognitive development. Recent work (Siegler, 2016; Pascual-Leone and Johnson, 2021) argued that, after the crisis of Piaget’s general theory and many years of fragmentation of cognitive developmental research, time is ripe for a new integration of cognitive developmental models across different domains. Our studies could be a contribution in that direction.

## Data Availability Statement

The raw data supporting the conclusions of this article will be made available by the authors, without undue reservation.

## Ethics Statement

Ethical review and approval was not required for the study on human participants in accordance with the local legislation and institutional requirements. Written informed consent to participate in this study was provided by the participants’ legal guardian/next of kin.

## Author Contributions

SP: conceptualization, methodology, investigation, data curation, statistical analyses, and writing. SM: conceptualization, methodology, statistical analyses, and writing. All authors contributed to the article and approved the submitted version.

### Conflict of Interest

The authors declare that the research was conducted in the absence of any commercial or financial relationships that could be construed as a potential conflict of interest.

## Footnotes

^1^The results of these studies should be interpreted with some caution. Some studies were designed to investigate the specific role of inhibition or working memory; however, until about 4 years of age, it is difficult to distinguish empirically the individual differences in the dimensions of working memory capacity and specific executive functions. Hence, some uncertainty remains on whether a specific function or a compound of them is involved in a specific task.

^2^The nesting is a consequence of a fact that a parameter, i.e., the correlation between two factors, is free in one model and fixed (at the value of 1.0) in the other. This method is widely used in research on executive function; e.g., see Miyake et al. (2000).

^3^A correlation between the errors of expressive vocabulary and expressive syntax is easily understandable, because of their shared method. The same holds of correct placements and appropriate marks, which measure two distinct aspects of the same responses. A positive correlation between the error components of spontaneous identifications and diffuse scribblings is perhaps surprising, but it could be understood considering the level of arousal: a child very excited and enthusiastic during the graphic activities will be more motivated to find missing elements, in case he/she is able to do so, or at least to scribble something, in case he/she is not able to do anything better.

^4^Instead, we did not test any model in which the direction of the effect between drawing and language was reversed, because the developmental priority of language on drawing renders *a priori* implausible that drawing has an effect on language development in this age range.
